# Sinus Pericranii (Parietal and Occipital) With Epicranial Varicosities in a Case of Craniosynostosis

**DOI:** 10.7759/cureus.21891

**Published:** 2022-02-04

**Authors:** Anchal Sharma, Monika Sharma

**Affiliations:** 1 Radiology, Maharishi Markandeshwar Medical College and Hospital, Solan, IND

**Keywords:** increased intracranial pressure, epicranial varicosities, sinus pericranii, oxycephaly, craniosynostosis

## Abstract

Sinus pericranii is a type of slow flow venous malformation with communication between intracranial venous system and epicranial veins through transosseous emissary veins. They can be isolated or may be seen with malformations like craniosynostosis. It has been postulated that transient intracranial venous hypertension in the late embryonic period could lead to the development of venous anomalies, including sinus pericranii. We present a case of oxycephaly with concurrent presence of sinus pericranii in parietal and occipital regions with epicranial varicosities with other imaging findings of raised intracranial pressure. Other findings suggestive of raised intracranial pressure were cerebellar tonsillar herniation/prominent optic nerve dural sleeves. The paucity of literature warrants future studies to establish role of intracranial hypertension in etiopathogenesis of sinus pericranii.

## Introduction

Sinus pericranii is a type of slow flow venous malformation with communication between intracranial venous system and epicranial veins through transosseous emissary veins. They can be acquired or congenital. If congenital, they may be seen with malformations like craniosynostosis. It has been postulated that transient intracranial venous hypertension in the late embryonic period could lead to the development of venous anomalies, including sinus pericranii. We present a case of oxycephaly with concurrent presence of sinus pericranii in parietal and occipital regions with epicranial varicosities. It is a very rare case scenario where there is concurrent presence of parietal as well as occipital sinus pericranii with presence of epicranial varicosities. Other findings suggestive of raised intracranial pressure were cerebellar tonsillar herniation and prominent optic nerve dural sleeves. The paucity of literature warrants future studies to establish the role of intracranial hypertension in etiopathogenesis of sinus pericranii.

## Case presentation

**Figure 1:**
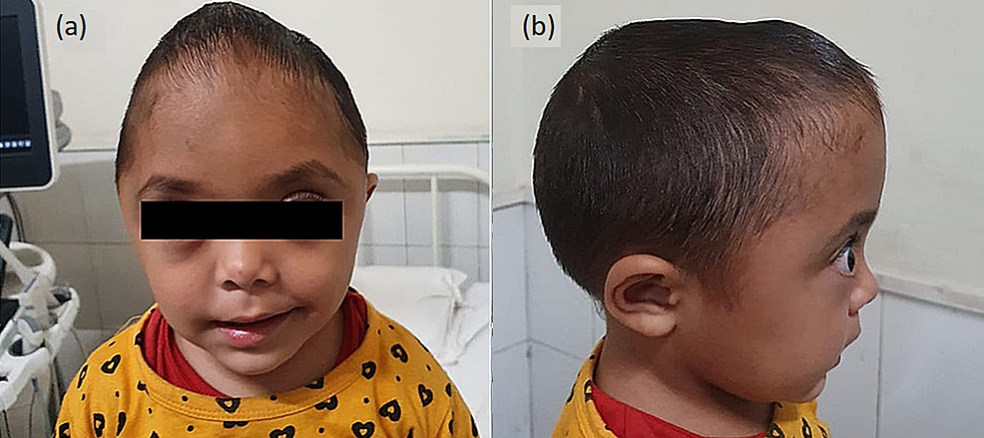
Clinical image showing abnormal skull shape with steeply rising forehead and dome-like apex at the site of anterior fontanelle (a) with exophthalmos (b).

A three-year-old girl child presented with painless pulsatile scalp swelling for one year. The swelling increased in size on crying and also on lying down. On examination, there was a swelling in parietal region with no discoloration of overlying skin. The child was seen to have abnormal skull shape with steeply rising forehead and dome-like apex at the site of anterior fontanelle with exophthalmos (Figures 1a, 1b). The anterior and posterior fontanelles were closed.

Born to non-consanguineous parents with no significant birth history, the patient’s height, weight, and milestones were age-appropriate. No neurological deficit was found. The child was sent for imaging which included x-ray skull lateral view, Doppler ultrasound, and MRI with MR venography, for further evaluation.

**Figure 2:**
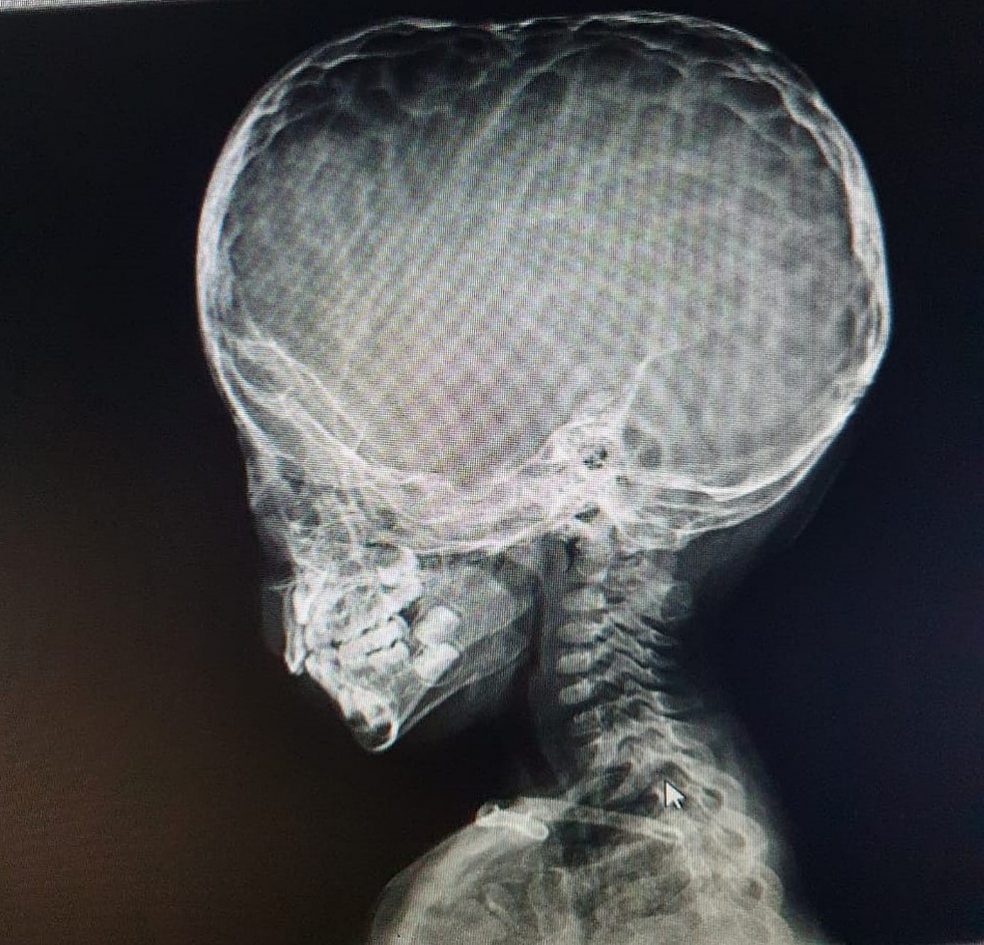
Skull radiography lateral view showing copper beaten appearance with widened roof of sella.

**Figure 3:**
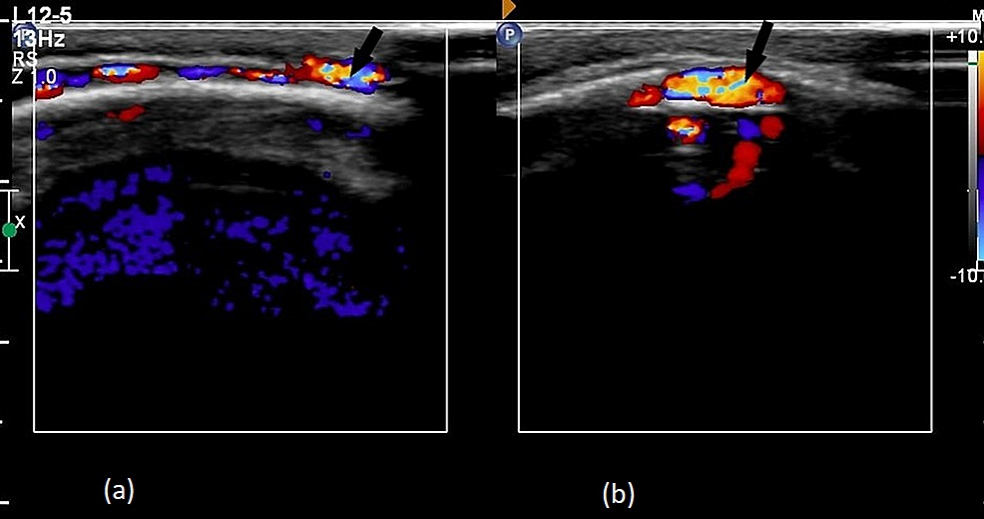
Color Doppler ultrasound showing slow venous flow in extracranial vessels in parietal region (black arrows) (a), in direct communication with superior sagittal sinus (b).

**Figure 4:**
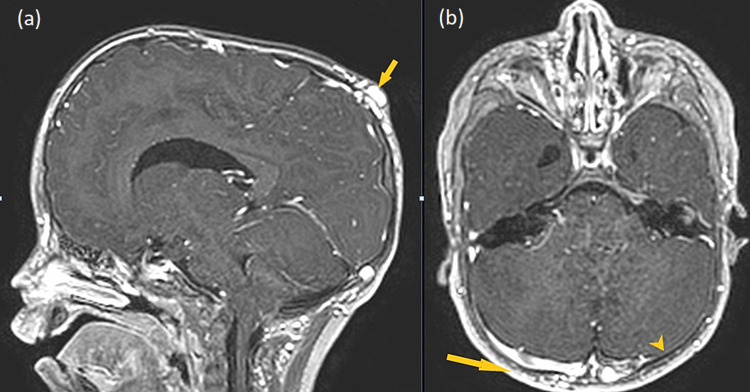
Sagittal (a) and axial (b) T1W post-contrast images. The images are showing prominent transcranial emissary veins traversing cranial defects in parietal and occipital regions, communicating with underlying superior sagittal sinus and torcular Herophili to supply scalp venous mass (yellow arrows). Hypoplastic left transverse sinus (yellow arrowhead). T1W: T1-weighted

On radiograph of skull (lateral view), there was classical copper beaten appearance with widened diaphragma sellae. All sutures were closed with abnormal skull shape, suggesting craniosynostosis (oxycephaly) (Figure 2). Color Doppler ultrasound showed an extracranial vascular structure in parietal region that communicated with intracranial vascular mass via feeding intraosseous vessels through a cranial defect (Figures 3a, 3b). On spectral analysis, venous flow was observed in the same lesion. Axial and sagittal T1-weighted (T1W) post-contrast images showed prominent emissary veins traversing cranial defects in the parietal and occipital regions receiving blood from underlying superior sagittal sinus and torcular Herophili, respectively; to supply the scalp venous mass (Figures 4a, 4b).

**Figure 5:**
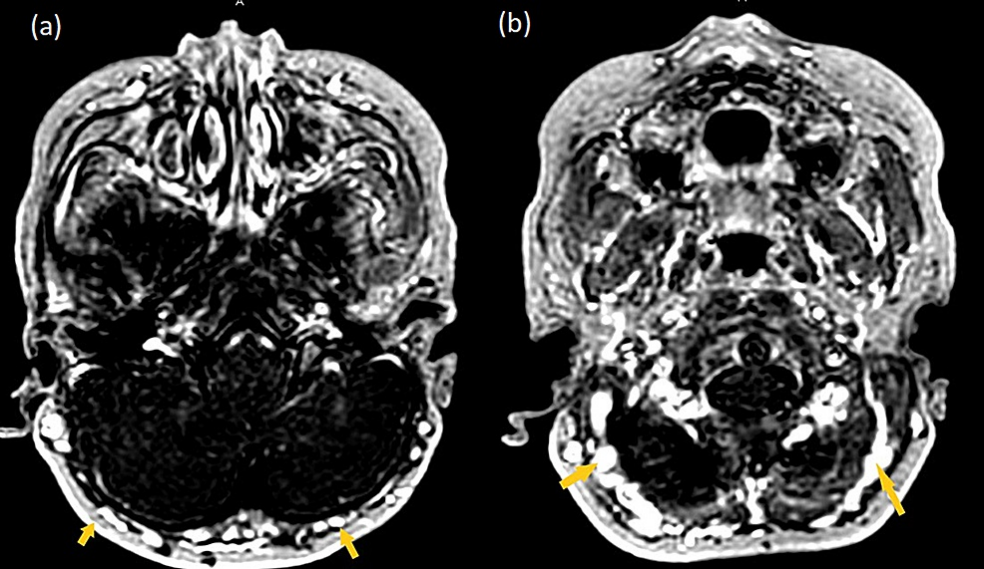
Axial T1W post-contrast images show epicranial varicosities involving bilateral occipital veins (yellow arrows) (a) and paravertebral venous plexus (yellow arrows) (b). T1W: T1-weighted

**Figure 6:**
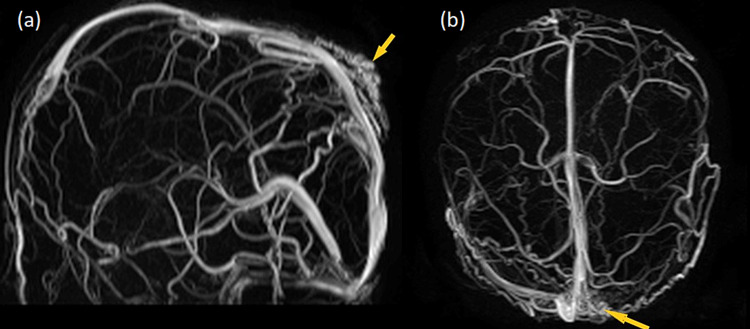
Three-dimensional time of flight MRV images. Lateral (a) and axial (b) images are showing transcranial veins communicating between intracranial venous system and extracranial scalp venous mass (yellow arrows). MRV: magnetic resonance venography

**Figure 7:**
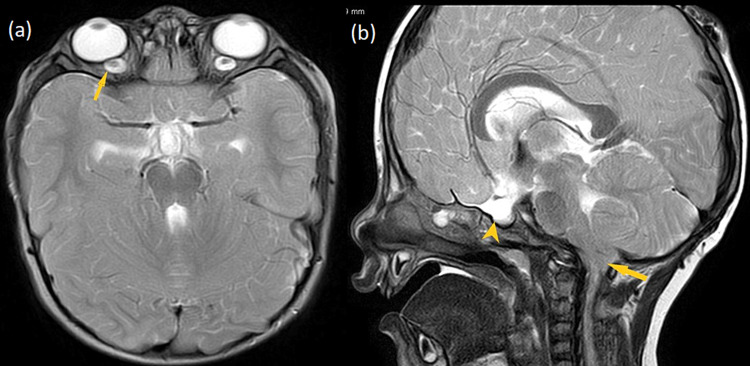
Axial (a) and sagittal (b) T2-weighted images. The images are showing prominent optic dural sleeves and flattening of optic discs (yellow arrow) (a), partially empty sella with widened diaphragma sellae (yellow arrowhead) and cerebellar tonsillar herniation (yellow arrow) (b). T2W: T2-weighted

There was marked dilatation and tortuosity noted involving bilateral occipital veins and paravertebral venous plexus in the region of the base of skull s/o epicranial varicosities (Figures 5a, 5b). Left transverse sinus was hypoplastic. The same findings were confirmed on MR venography (Figures 6a, 6b). There was no e/o of thrombosis. MR angiography was normal as well. Other notable findings suggesting raised intracranial pressure were prominent optic dural sleeves with flattening of optic discs, cerebellar tonsillar herniation, and partially empty sella with widened diaphragma sellae (Figures 7a, 7b). The parents denied surgical intervention so follow-up was advised. Also, the parents were educated about dangerous signs like persistent headache, nausea, vertigo, blurring of vision and told to seek medical help immediately.

## Discussion

Craniosynostosis or craniostenosis is the premature fusion of cranial sutures. It can be simple (involving only one suture) or compound (where two or more sutures are involved) [1,2]. The birth prevalence of craniosynostosis ranges from 3.1 to 4.8 per 10,000 live births [3-6]. Oxycephaly is rare compound craniosynostosis due to combination of severe sagittal and coronal synostoses. The typical oxycephalic head is abnormally high, with a steeply rising forehead and a blunt, dome-like apex at the site of the anterior fontanel as in our case. The glabella, supra-orbital ridges, and frontal eminences are flattened out. There is usually a widening of the transverse and a narrowing of the anteroposterior diameter of the skull. Exophthalmos is a common feature due to shortening of the anteroposterior diameter of the orbits. This is frequently associated with a divergent squint. This condition may result in microcephaly with raised intracranial pressure and neurologic impairment [7].

The interesting finding in our patient is the concurrent presence of sinus pericranii (parietal and occipital regions) and epicranial varicosities in oxycephalic craniosynostosis. On review of literature, no such case has been reported so far. Since Stromeyer used the term "sinus pericranii" in his report in 1850, approximately 170 cases have been reported suggesting its rare occurrence. More so, few cases of sinus pericranii have been reported in association with craniosynostosis as in our case [7]. Sinus pericranii is a rare, benign, venous anomaly consisting of an emissary intradiploic vein deriving from an intracranial sinus, with an increased subgaleal drainage composed of a network of thin-walled veins that form varix on the external table of the skull. The anastomotic connections may consist of either a single transosseous vessel or multiple venous structures, which in rare cases can course within the skull bones for several centimeters, causing extensive diploic erosion [8,9].

Sinus pericranii can be acquired or congenital or associated with other malformations (i.e., secondary), including craniosynostosis/craniosynostosis syndromes like Crouzon disease and Apert syndrome [10], or intracranial venous abnormalities such as dural sinus hypoplasia [9]. The sinus pericranii is frequently associated with intracranial developmental venous anomalies suggesting a congenital predisposition. Patients are usually asymptomatic or present with scalp mass, headache, vertigo, and nausea. Treatment is usually undertaken for cosmetic reasons and rarely for complications, such as hemorrhage, thrombosis, emboli, and infection [11,12]. Clinical diagnosis may be difficult but imaging appearance is usually characteristic. Treatment options include surgical resection or transvenous endovascular approach; however, if the sinus pericranii is dominant (i.e., if drainage of the brain is through sinus pericranii, bypassing the usual venous outlets), then treatment is avoided [11]. Our case was kept on follow-up under close observation to be treated later if the clinical condition worsened.

## Conclusions

It is a historically known fact that craniosynostosis is accompanied by raised intracranial pressure which is further corroborated by the roentgen copper beaten skull appearance as seen in our case, but the presence of sinus pericranii and epicranial varicosities as a consequence of raised intracranial pressure in craniosynostosis is yet to be established. It is a historically known fact that craniosynostosis is accompanied by raised intracranial pressure which is further corroborated by the roentgen copper beaten skull appearance as seen in our case, but the presence of sinus pericranii and epicranial varicosities as a consequence of raised intracranial pressure in craniosynostosis is yet to be established. On review of literature, no study was found establishing the etiopathogenesis of sinus pericranii in craniosynostosis. In our case, there were many imaging features s/o raised intracranial pressure, such as copper beaten skull appearance on radiograph, cerebellar tonsillar herniation, prominent optic dural sleeves, and partially empty sella with widened diaphragma sellae. The idea to report this case is not only its unusual and rare presentation but also to understand its etiopathogenesis.
